# PR depression with multilead ST elevation and ST depression in aVR by left circumflex artery occlusion: How to differentiate from acute pericarditis

**DOI:** 10.1111/anec.12752

**Published:** 2020-02-21

**Authors:** Zhong‐Qun Zhan, Kjell Nikus, Yochai Birnbaum

**Affiliations:** ^1^ Department of Cardiology Shenzhen Hospital‐University of Chinese Academy of Sciences Shenzhen China; ^2^ Department of Cardiology Heart Center Tampere and Faculty of Medicine and Health Technology Tampere University Hospital Tampere University Tampere Finland; ^3^ The Section of Cardiology Baylor College of Medicine and Texas Heart Institute Luke Medical Center Houston TX USA

**Keywords:** acute myocardial infarction, left circumflex artery, PR segment

## Abstract

PR‐segment depression with multilead ST‐segment elevation and ST‐segment depression in lead aVR are classic ECG manifestation of acute pericarditis. We present a patient, where the etiology of these ECG features was acute ST‐elevation myocardial infarction due to left circumflex artery occlusion. To avoid misdiagnosis, unnecessary examinations, and inappropriate therapeutic decisions, the possibility of ST‐segment elevation myocardial infarction should be kept in mind even when ECG changes typical for pericarditis are encountered in chest pain patients. Findings of QRS widening and QT interval shortening in leads with ST‐segment elevation could help to differentiate acute ST‐segment elevation myocardial infarction from acute pericarditis.

## 1 CASE REPORT

**Figure 1:**
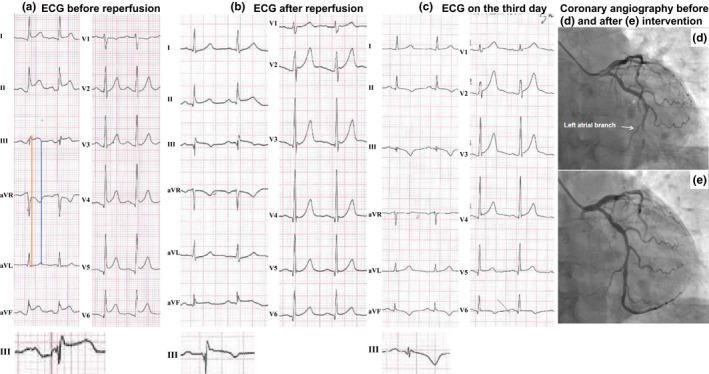
(a): The ECG on arrival shows multilead ST‐segment elevation (I, II, III, aVF, and V4‐V6), ST‐segment depression in lead aVR, and PR‐segment depression in leads II, III, aVF and V2‐V6 with concomitant PR‐segment elevation in lead aVR. Notched P wave is present in lead III. The P‐wave width is 129 ms. The amplified tracing in lead III at the bottom clearly shows the notched and widened P wave. The red line at the J‐point indicates that the QRS duration in lead III is longer than in lead aVL. The blue line at the termination of the T wave indicates that the QT interval in lead III is shorter than in lead aVL. (b) The postintervention ECG shows minor ST‐segment elevation (II, III, and aVF), and disappearance of PR‐segment deviation. The amplified tracing in lead III at the bottom clearly shows disappearance of the notched and widened P wave. (c) The ECG on the third day in hospital shows complete ST resolution and prominent negative T waves in the inferior leads. The P‐wave width is 110 ms. (d) Coronary angiography shows total occlusion of the left circumflex coronary artery just after the take‐off of the left atrial branch (white arrow). (e) Coronary angiography shows patent left circumflex coronary artery after intervention

A 49‐year‐old human was admitted to the emergency department with persistent chest pain radiating to the left arm and diaphoresis for 40 min. Coronary artery disease risk factors included smoking and a sedentary life style. His blood pressure was 131/99 mmHg, and the heart rates were 100 bpm. The heart sounds were normal without murmurs or a friction rub, and lung auscultation revealed no rales. The first electrocardiogram (ECG) showed multilead ST‐segment elevation (I, II, III, aVF, and V4‐V6), ST‐segment depression in lead aVR, and PR‐segment depression in leads II, III, aVF, and V3‐V6 with concomitant PR‐segment elevation in lead aVR (Figure 1a). Notched P wave was present in lead III. P‐wave width was 129 ms, and QRS width was 99 ms (Figure 1a). Troponin I level was 0.020 ng/ml (normal value <0.040 ng/ml). NT‐proBNP level was <60 pg/ml (normal value <300). There was a suspicion of acute ST‐elevation myocardial infarction (STEMI), and the patient received ticagrelor 180 mg, acetylsalicylic acid 300 mg, and rosuvastatin 20 mg immediately.

Taking into account the PR‐segment deviations and absence of reciprocal ST‐segment depression, the consultant cardiologist could not rule out acute pericarditis and a bedside echocardiography was performed showing inferolateral hypokinesia without pericardial effusion. Emergency coronary angiography revealed acute occlusion of the distal left circumflex coronary artery (Figure 1d) and 70% stenosis in the proximal right coronary artery. White thrombus was aspirated, and a stent was implanted into the left circumflex coronary artery. TIMI 3 blood flow was obtained (Figure 1e), and chest pain was completely relieved after the procedure. The door‐to‐balloon time was 71 min. The postintervention ECG showed minor ST‐segment elevation (II, III, and aVF) and disappearance of PR‐segment deviation. The P‐wave notching disappeared, and the P‐wave width was 120 ms (Figure 1b). The patient recovered quickly and was discharged home on the fourth day. The ECG on the third day after the intervention showed complete ST resolution and prominent negative T waves in the inferior leads and lead V6. The R/S ratio in lead V1 was >1 indicating lateral myocardial infarction. The P‐wave duration was 110 ms (Figure 1c).

## 2 DISCUSSION

Obviously, acute occlusion of the left circumflex artery can perfectly explain the ST‐segment elevation in leads I, II, III, aVF, and V4‐V6, with concomitant ST‐segment depression in lead aVR. The following ECG evolution (B and C) including resolution of the ST‐segment deviation and negative T waves in the inferior leads confirmed the diagnosis of STEMI. Interestingly, the ECG manifestation of PR‐segment depression in leads II, III, aVF, and V3‐V6 with concomitant PR‐segment elevation in lead aVR together with diffuse ST‐segment elevation in 7 leads (I, II, III, aVF, V4, V5, V6) and absence of reciprocal ST‐segment depression in the right precordial leads raised a suspicion of concomitant acute pericarditis. However, the facts that the PR‐segment deviation completely disappeared and the chest pain was completely relieved after successful reperfusion therapy made this possibility untenable. Left atrial infarction is one plausible explanation for the ECG changes (Lu, De Venecia, Patnaik, & Figueredo, 5). On angiography, the side branch to the left atrium was just proximal to the occlusion, and it could be that the thrombus was more proximal covering the side branch ostium in the acute phase. There was a 1‐hr time delay between the ECG and the angiography, and the patient received antithrombotic therapy immediately after the ECG recording.

In the large necropsy study by Cushing, Feil, Stanton, and Wartman (1942), they reported a 17% incidence of atrial infarction in patients with ventricular infarction. The diagnosis of atrial infarction depends largely on ECG presentations. Although no consistent ECG abnormalities were reported, common ECG abnormalities (Lu et al., 5) include PR‐segment depression or elevation, morphologic P‐wave changes such as notching, increase in amplitude, and transient changes in contour and arrhythmias. The change in P‐wave duration from 129–110 ms within a short time combined with the PR‐segment changes and the notched P wave seems to support the hypothesis of atrial involvement of the infarct process in our case. There were changes in the morphology and duration of the P wave which also diminished after coronary reperfusion. These changes together with the PR‐segment displacement suggest atrial myocardial ischemia due to transient obstruction of left atrial branch tributary of the LCX. Other possible explanations for morphologic changes of the P wave and the PR‐segment deviations include increased sympathetic activity, atrial dilatation, or intra‐atrial block etc (Lu et al., 5).

## 3 HOW TO DIFFERENTIATE FROM ACUTE PERICARDITIS AND EARLY REPOLARIZATION WITH ST ELEVATION

PR‐segment depression with multilead ST‐segment elevation and ST‐segment depression in lead aVR is a typical ECG presentation in acute pericarditis (McNamara, Ibrahim, Satti, Ibrahim, & Kiernan, 6). In patients with STEMI, the ST‐segment elevation is found in leads overlying the transmural ischemic region, and reciprocal ST‐segment depression is often found in leads related to distant nonischemic regions (Kudenchuk, Maynard, & Cobb, 4). Compared to patients in the STEMI group, patients in the pericarditis group have: (a) greater PR‐segment elevation and deeper ST‐segment depression in lead aVR; (b) higher number of leads with ST‐segment elevation and PR‐segment depression; and (c) lower number of leads with reciprocal ST‐segment depression (Rossello, Wiegerinck, & Alguersuari, 8). The ECG variables that support the diagnosis of pericarditis with a sensitivity of 85.9% and a specificity of 85.3% include the following: PR‐segment elevation in lead aVR, ST‐segment depression at the J‐point ≥0.05 mV in lead aVR, ≥7 leads with ST‐segment elevation, ≤1 lead with ST‐segment depression, and ≥0 leads with PR‐segment depression (Rossello et al., 8). In acute pericarditis, PR‐segment depression was most often found in both the anterior and inferior leads, while depressions in the lateral leads were less common. The combination of PR‐segment depressions in both precordial and limb leads had the most favorable predictive power to differentiate acute pericarditis from STEMI (positive 96.7% and negative power 90%) (Porela, Kytö, Nikus, Eskola, & Airaksinen, 7).

Interestingly, the ECG (Figure 1a) of our patient case fulfills the typical presentations of the above‐mentioned criteria in acute pericarditis. To our knowledge, we are the first to report a case of PR‐segment depression with multilead ST‐segment elevation and ST‐segment depression in lead aVR caused by left circumflex artery occlusion. However, patients with acute STEMI, but not those with acute pericarditis, often present with widening of the QRS complex and transient shortening of the QT interval in leads with ST‐segment elevation. QRS duration ≤70 ms for the leads with maximal ST‐segment elevation and QT dispersion ≤63 ms are significant criteria to aid in the diagnosis of acute pericarditis (Rossello et al., 8). These new findings may improve the differential diagnostic yield of the classical ECG criteria. In our patient case, the QRS duration was longer and the QT interval shorter in lead III than in lead aVL (see the red and blue lines in Figure 1a) supports the diagnosis of STEMI.

Of note, profound PR‐segment depression ≥1.2 mm in the inferior leads in patients with inferior STEMI was associated with high risk for the development of atrioventricular block, supraventricular arrhythmias, and cardiac free‐wall rupture (Jim et al., 3). Our patient case with a favorable outcome may be related to quick reperfusion.

Diffuse ST‐segment elevation in the inferolateral leads associated with ST‐segment depression in aVR and even with PR‐segment depression is commonly found in early repolarization with ST elevation (Birnbaum, Perez Riera, & Nikus, 1). In this condition, there should be no clinical symptoms or signs of acute pericarditis or STEMI (Birnbaum et al., 1).

## 4 CONCLUSION

Transient PR‐segment depression with multilead ST‐segment elevation and ST‐segment depression in lead aVR can be caused by left circumflex artery acute occlusion. The possibility of STEMI should be kept in mind even when ECG changes typical for pericarditis are encountered in chest pain patients to avoid misdiagnosis, unnecessary examinations, and inappropriate therapeutic decisions. Specific new findings of QRS complex widening and QT interval shortening in leads with ST‐segment elevation could help to differentiate acute STEMI from acute pericarditis.

## CONFLICT OF INTEREST

All authors have reported that they have no relationships relevant to the contents of this article to disclose.

## AUTHOR CONTRIBUTION

All authors have made substantive contributions to the study. Zhan ZQ reported the case and drafted the manuscript. Nikus K and Birnbaum Y explained the case and revised the manuscript. All authors endorse the data and conclusions.

## ETHICS

This case report was approved by the *Shenzhen Hospital ‐ University of Chinese Academy of Sciences*. Patient had the opportunity to read the present case report and had no objections to the case report.
